# Comment on Menzel et al. Common and Novel Markers for Measuring Inflammation and Oxidative Stress Ex Vivo in Research and Clinical Practice—Which to Use Regarding Disease Outcomes? *Antioxidants* 2021, *10*, 414

**DOI:** 10.3390/antiox10060836

**Published:** 2021-05-24

**Authors:** Simona Mrakic-Sposta, Maristella Gussoni, Michela Montorsi, Alessandra Vezzoli

**Affiliations:** 1 National Research Council—Institute of Clinical Physiology (CNR-IFC), 20162 Milan, Italy; alessandra.vezzoli@cnr.it; 2 National Research Council—Institute of Chemical Sciences and Technologies “G. Natta”-SCITEC (CNR-SCITEC), 20133 Milan, Italy; maristella.gussoni@unimi.it; 3 Department of Human Sciences and Promotion of the Quality of Life, San Raffaele Roma Open University, 20122 Milan, Italy; michela.montorsi@uniroma5.it

Recently, Menzel A et al. published a review titled “Origin and Physiological Aspects of Oxidative Stress (OS), inflammation and markers of OS, relation to disease and practical aspects” [1].

We greatly appreciated this review, which aimed not only at promoting, but also at advancing scientific knowledge in the field of OS and inflammation research. Indeed, the authors addressed many topics, shedding light on markers of primary inflammation agents, direct markers of ROS, as well as indirect markers of oxidative damage, antioxidant enzymes and endothelial markers, and transcription factors. At the same time, emphasis was given to the role played by advanced techniques, such as mass spectrometry, Nuclear Magnetic and Paramagnetic Resonance (NMR, EPR), suitable for performing these measurements.

However, in regard to the last technique, EPR, the authors claim as follows: “Regarding OS, ROS may be measured directly, […] but this is usually not realized in clinical practice or even research due to equipment restrictions and sample instability”. Again, a few pages later: “However, due to the difficulties regarding the need to measure fresh samples and the marginal availability of EPR instruments, this method has not found many clinical or even research applications. […] In an interesting but small-scale study with 100 middle-aged subjects, capillary blood was measured by EPR, following the addition of a spin probe. […] EPR measures correlated well with PCs and TBARS.”

Following these claims, we would like to add some remarks with the purpose of shedding further light on this matter.

Widely reported and recognized, EPR is a non-invasive technique, suitable for a direct and quantitative measure of ROS. It finds many fields of application, among which is medicine [2,3,4,5,6,7,8]. Here, it can be used both for paramagnetic species and free radical studies in so-called biologically active materials (e.g., blood, tissues) [2,9].

Indeed, EPR is capable of returning ‘intrinsic’ quantitative information of free radical levels. The recorded absorption spectra can provide a direct detection of the “instantaneous” presence of free radical species in the sample [2,9]. Moreover, the technique usually plays a major role in the assessment of most of the oxidants characterized by a very short half-life (ns, μs) by adopting spin-traps/probes [10]. Finally, EPR allows us to obtain kinetical data and to follow a clinical treatment. Indeed, fresh samples are not necessarily required for data collection; frozen samples can be adopted to study entire blood or its components, such as urine, saliva, etc. [11]. Dedicated EPR instruments are also available for particular clinical applications.

We are grateful to the authors for having cited our publication in their review [12], which, as they report, was really a small-scale study. Since then, however, more than 1000 samples have been the object of publication by our research group, in collaboration with others, in healthy and pathological conditions, such as neurodegenerative, metabolic, immune diseases; supplementation diet and therapy monitoring, state/events, such as hypoxia e hyperoxia, and exercise [4,5,6,13].

At the same time, it was, and up to now still is, encouraging the good correlation found between EPR data ‘directly’ detected and indirect markers (i.e., protein carbonyl). Indeed, EPR technique was also found to be suitable for other measurements, such as antioxidant capacity (TAC), and nitric oxide (NO).

Finally, we want to emphasize the potentialities of this technique, which we are confident will be designed to achieve growing development in the field of research and clinical medicine. Additionally, correlations between EPR measurements and those obtained adopting different methods (immune/histochemical assay, HPLC, NMR) could help to elucidate the kinetic and molecular mechanisms of OS in different physiological, paraphysiological, and pathological conditions.
